# Special Issue “Nanoparticle-Mediated Drug Delivery, Imaging, and Control of Cellular Functions”

**DOI:** 10.3390/ph16101344

**Published:** 2023-09-22

**Authors:** Okhil K. Nag, James B. Delehanty

**Affiliations:** Center for Bio/Molecular Science and Engineering, Naval Research Laboratory (NRL), Code 6900, 4555 Overlook Ave. SW, Washington, DC 20375, USA; james.delehanty@nrl.navy.mil

Over the past several decades, nanoparticles (NPs) have shown promising capabilities in the field of medicine for their applications as vehicles for targeted drug delivery. NPs have also shown utility in biological imaging, sensing, and disease diagnosis [1,2,3,4,5]. More recently, NPs have been used to control cellular function by regulating cell–cell signaling, modulating protein expression, and even influencing genetic expression [6,7]. In this Special Issue, entitled “Nanoparticle-Mediated Drug Delivery, Imaging, and Control of Cellular Functions”, we aim to highlight some new advances in NP-related contributions in the field of medicine. Briefly, this Special Issue provides an overview of diverse potential applications with various types of NPs, such as polymeric NPs, semiconducting quantum dots (QDs), gold NPs (AuNPs), and magnetic NPs. This Special Issue includes a total of 10 articles, including a comprehensive review article highlighting very recent advances in NP applications in the treatment of cardiovascular disease. Two further reviews discuss the use of NPs in dentistry and transgene delivery to human-induced pluripotent stem cells.

While all the contributions to this Special Issue are interesting and will play a significant role in advancing this field, we briefly focus on some of these studies here. Safwat et al. fabricated gold NPs for the delivery of Epigallocatechin-3-gallate (EGCG), a pleiotropic compound with anticancer, anti-inflammatory, and antioxidant properties, to treat Ehrlich ascites carcinoma in mouse models [8]. With an excellent EGCG loading efficiency (93%), this formulation significantly reduces the size of Ehrlich ascites tumors in mouse models. In a tumor-targeted approach with arginine-glycine-glutamic acid (RGD), Itzhak et al. presented proteinoid nanocapsules (NCs) loaded with a combination of Palbociclib (Pal) and Alpelisib (Alp), which primarily bind with CDK4/6 and P13K receptors, respectively, and play critical roles in synergistically inhibiting the growth, proliferation, and motility of tumor cells [9]. These NCs were demonstrated to treat various human cancer cell lines (e.g., MCF7 (breast adenocarcinoma), HCT116 (colon carcinoma) and A549 (lung carcinoma), a patient-derived xenograft (PDX) tumor spheroid model, and an in vivo (mouse) PDX tumor model. In a different direction, Šafranko et al. presented multifunctional nitrogen-doped carbon quantum dots (CQDs) as antioxidant, antitumor, and Fe^3+^ ion-sensing agents [10]. In this study, we prepared and studied three different CQDs via different methods. For instance, we examined pure CQDs without the use of N-doping (pristine CQDs), CQD@Gly using N-dopant Gly, and CQD@Arg using Arg as an N-dopant with a higher nitrogen content compared to Gly. The antioxidant properties of CQD@Arg (EC_50_ = 39.21 ± 0.41 µg/mL) were determined to be about twofold that those of pure CQDs (EC_50_ = 79.83 ± 1.72 µg/mL). In this Special Issue, we reported a fabrication and application of a fluorescently trackable (perylene, PY) liquid crystal nanoparticle (PY-LCNP) as a platform for the multivalent display and delivery of a low-molecular-weight antioxidant, such as 2,2,6,6-tetramethylpiperidine-N-oxyl (TEMPO), as an ROS scavenger to live cells [11]. The conjugate showed excellent cellular uptake and efficacy in reducing ROS activity and lipid peroxidation when the cells were challenged with the ROS-generating agents H_2_O_2_ and tBHP. In human cervical adenocarcinoma (HeLa) cells exposed to extracellular ROS-producing H_2_O_2_, the PY-LCNP-PEG/TEMPO conjugate showed a >300% enhancement of ROS scavenging activity and a 26% reduction in lipid peroxidation compared to cells incubated with free TEMPO (TEMPO_free_) in bulk solution.

In a review article in this Issue, Moraes et al. presented a comprehensive review of the application of NPs in the diagnosis, prevention, and treatment of many oral diseases, including dental caries, periodontal diseases, pulp and periapical lesions, oral candidiasis, denture stomatitis, hyposalivation, and head, neck, and oral cancer [12]. The authors underscore the disease-specific application of various NPs, including organic polymeric NPs and inorganic metal oxide NPs. In another excellent review, Manners et al. surveyed multimodal applications of NPs in theranostic nanomedicines to treat cardiovascular diseases [13]. This review highlighted the current strategies, future perspectives, and challenges of the NP-mediated diagnosis and treatment of thrombosis obstructs, atherosclerosis, hyperlipidemia, hypertension, pulmonary arterial hypertension, and ischemic stroke. Finally, Yamoah et al. reviewed recent NP-mediated transgenic gene delivery to human-induced pluripotent stem cells (hiPSCs) [14]. The authors highlighted the advantages of NP-mediated transgene delivery over other methods, including high efficiency, low cytotoxicity, biodegradability, low cost, directional and distal controllability, and efficient in vivo application.

Overall, this Special Issue successfully assembles very recent advances in the application of NPs in the field of medicine encompassing bioimaging, biosensing, disease diagnoses, and treatment. We believe that the contributions in this Special Issue will draw in wide groups of readers and strongly encourage the development of new approaches and strategies for the fabrication of NP-mediated systems to advance the field of medicine. Finally, the Guest Editors would like to thank all the authors and reviewers for their valuable contributions to this Special Issue. We would also like to thank MDPI for hosting this Special Issue and Ms. Amber Zhao for her kind assistance.
